# Clinicopathological and prognostic significance of HER-2/neu and VEGF expression in colon carcinomas

**DOI:** 10.1186/1471-2407-11-277

**Published:** 2011-06-27

**Authors:** Qingguo Li, Daorong Wang, Jing Li, Ping Chen

**Affiliations:** 1 Department of General Surgery, First Clinic Medical School of Yangzhou University, Yangzhou, China; 2 Department of Medical Oncology, Second Clinic Medical School of Yangzhou University, Yangzhou, China

## Abstract

**Background:**

HER-2/neu and VEGF expression is correlated with disease behaviors in various cancers. However, evidence for their expression in colon cancer is rather contradictory both for the protein expression status and prognostic value. HER-2/neu is found to participate in VEGF regulation, and has known correlation with VEGF expression in some tumors. In this study, we investigated HER-2/neu and VEGF expression in Chinese colon patients and explored whether there was any correlation between their expression patterns.

**Methods:**

HER-2/neu and VEGF were investigated immunohistochemically using tumor samples obtained from 317 colon cancer patients with all tumor stages. Correlation of the degree of staining with clinicopathological parameters and survival was investigated.

**Results:**

Positive expression rates of HER-2/neu and VEGF in colon cancer were 15.5% and 55.5% respectively. HER-2/neu expression was significantly correlated with tumor size and distant metastases (*P *< 0.05), but was not an independent prognostic marker of survival *(P > 0.05)*. Expression of VEGF was significantly correlated with tumor size, tumor stage, lymph node metastases, and distant metastases (*P *< 0.05). The 5-year survival rate in patients with negative and positive VEGF expression was 70.2% and 61.9% respectively; the difference was not statistically significant *(P = 0.146)*. No correlation between HER-2/neu and VEGF expression was detected (*P = *0.151).

**Conclusions:**

HER-2/neu and VEGF are not important prognostic markers of colon cancer. The present results do not support any association between HER2/neu and VEGF expression in this setting.

## Background

Colorectal cancer (CRC) is one of the most common malignancies worldwide and is the fourth leading cause of cancer-related death in China. Although the development of new cytotoxic agents such as oxaliplatin and irinotecan and new surgical techniques has improved prognosis of CRC, once patients develop resistance to chemotherapeutic regimens no other treatment options are available. Recently, therapeutic strategies have been improved by the development and availability of monoclonal antibodies. Investigators have recently been evaluating biologic and molecular targets for their possible roles as prognostic markers and as targets for therapy.

The HER-2/neu oncogene, also known as c-erbB-2, encodes a transmembrane tyrosine kinase receptor, homologous to epidermal growth factor receptor (EGFR), This receptor is involved in the growth and progression of malignant cells. For instance, overexpression of the HER-2/neu is detectable in 25%-35% of breast cancers [1-3]. Treatment of these patients with trastuzumab (Herceptin), an anti-HER-2/neu monoclonal antibody, has been shown to reduce tumor volume, magnify the effects of chemotherapy, and increase survival rate in primary and metastatic breast cancer [4,5]. The success of anti-HER-2/neu therapy in breast cancer has led to evaluations of HER-2/neu expression in multiple tumors, colon cancer among them, but conflicting data exist with reports of its expression in CRC ranging at 0-80% [6-8].

Vascular endothelial growth factor (VEGF)-A, formerly known as VEGF, is an angiogenic factor that is produced by tumor cells to stimulate intratumoral microvessel proliferation (neoangiogenesis). Tumor angiogenesis contributes to the metastatic process by promotion of leaking blood vessels for vascular invasion [9,10]. VEGF expression has been shown to be upregulated in many tumors and to be of prognostic value [11-13]. Furthermore, treatment with bevacizumab, a monoclonal antibody against VEGF, in combination with chemotherapy results in significant improvements in overall survival and progression-free survival compared with chemotherapy alone [14-16]. In addition, recently HER-2/neu was found to participate in and be correlated with VEGF expression [17-20]. However, no previous studies have clarified their associations in colon cancer. The purposes of this study were to examine expression status of HER-2/neu and VEGF in colon cancer and to evaluate whether their expression levels are correlated with each other and with clinicopathological parameters and prognosis.

## Methods

Between January 2000 and December 2005, a total of 317 patients received elective surgery for colon cancer at the Department of General Surgery of First Clinic Medical School of YangZhou University. The patients comprised 187 men and 130 women aged 21-86 (mean 57.8) years. No patient received anticancer treatment prior to surgery. Patients' clinicopathologic parameters including sex, age, and tumor differentiation, location, and pTNM pathological classification according to the International Union Against Cancer (UICC) were collected.

### Immunohistochemistry

Immunohistochemical analysis for HER-2/neu and VEGF expression was performed on formalin-fixed paraffin-embedded sections of surgical specimens. The slides were deparaffinized in xylene and rehydrated in ethanol solution. Endogenous peroxidase was blocked with 0.3% H_2_O_2 _in methanol for 10 min. The slides were immersed in 10 mm citric buffer (pH 6.0) with heating for 15 min for antigen retrieval, then cooled at room temperature for 20 min and washed with phosphate-buffered saline (PBS). Nonspecific binding was blocked by preincubation with 10% fetal calf serum in PBS with 0.01% sodium azide then the slides were incubated in a humidifier chamber for 1 h with antibody against VEGF(titer 1:50; Dako Cytomation, Denmark) and HER-2/neu HercepTest™ kit (titer 1:100;Dako Cytomation, Denmark). After washing three times in PBS, the slides were incubated with the envision-HrP complex (undiluted; Dako) for 60 min then visualized with diaminobenzidine and counterstained with hematoxylin. For substitute negative controls, the primary antibody was replaced with PBS. Positive controls included breast cancer tissue known to exhibit high levels of marker.

### Scoring systems

The slides were assessed by two pathologists with minimal interobserver variability including resolution of observed differences by simultaneous reevaluation. HER-2/neu staining was scored semiquantitatively according to the following scoring system approved by the US Food and Drug Administration (FDA): 0, no immunostaining or membrane staining in < 10% of the tumor cells; 1+, incomplete membrane staining of > 10% of tumor cells; 2+, weak-to-moderately complete membrane staining of > 10% of tumor cells; 3+, a moderate-to-strongly complete membrane staining of > 10% of tumor cells. Scores of 0 or 1+ indicated a tumor negative for HER-2/neu expression, and scores of 2+ and 3+ were regarded as positive expression of HER-2/neu.VEGF was graded according to previously established criteria [21]. A positive result constituted > 10% of cells stained, and a negative result constituted < 10% of cells stained. Intensity of staining was graded on a scale of 0 to 3+, with 3+ representing the strongest staining.

### Follow-up

Patients underwent continuous follow-up as of January 2011. No patient was lost to follow-up. The median follow-up interval was 68.8 months.

### Statistics

The results were analyzed using the statistical software package SPSS for Windows, version 13 (SPSS, USA). Association of HER-2/neu and VEGF staining with clinicopathological parameters was analyzed by χ2 test. Overall survival curves were calculated using the method of Kaplan and Meier, differences between the curves were analyzed by log-rank test. Statistical significance was set at *P < 0.05*.

## Results

### Expression of HER-2/neu and VEGF

**Figure 1:**
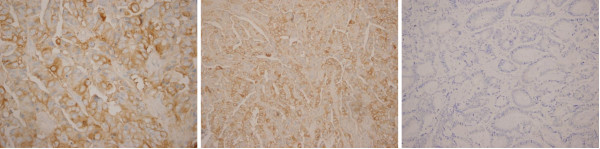
**Immunohistochemical staining for HER-2/neu and VEGF (original magnification, × 200)**. (a) Strong cytoplasmic stain in > 10% of tumor cells. (b) VEGF stained in > 10% of tumor cells. (c) No expression of VEGF and c-erbB-2 in colon cancer.

HER-2/neu expression was positive in 49 of 317 colon cancer samples (15.5%) and negative in the remaining 268 samples (84.5%), only seven samples were strongly positive. VEGF expression was positive in 176 of 317 colon cancer samples (55.5%) and negative in the remaining 141 samples (44.5%), 46 samples(14.5%) were 1+,79(24.9%) were 2+, and 51(16.1%) were 3+. Figure 1 shows HER-2/neu and VEGF staining patterns in colon cancer.

### Association between HER-2/neu and VEGF

HER-2/neu was detectable in 31 of 176 (17.6%) patients with VEGF-positive expression, in contrast to 18 of 141 (12.8%) patients with VEGF-negative expression (Table 1); the difference was not statistically significant.

**Table 1:** Relation between HER-2/neu and VEGF expression

VEGF	expression	HER-2/neu	expression	Total	***P-*value**^**a**^
				
		Positive	Negative		
Positive		31	145	176	

Negative		18	123	141	0.151

^a ^Pearson χ^2 ^test

### Correlation of molecular markers and clinicopathological features

Correlations of HER-2/neu and VEGF in colon cancer with clinical features are shown in Table 2. HER-2/neu expression correlated with tumor size and distant metastases (both *P *< 0.05), but not with other clinicopathological features assessed (all *P > 0.05*). Expression of VEGF correlated with tumor size, T stage, lymph node metastases and distant metastases *(*all *P *< 0.05). Statistical evaluation of VEGF expression according to age, sex, tumor location, differentiation grade, and perineural invasion revealed no significant difference among these variables (all *P > 0.05*).

**Table 2:** Clinicopathological variables and their correlation with immunohistochemical expression of HER-2/neu and VEGF

Variable	Patients	HER-2/neu		***P*-value**^**a**^	VEGF		***P*-value**^**a**^
					
		Positive	Negativ		Positive	Negative	
Sex				0.553			0.438
Male	187	29	158		105	82	
Female	130	20	110		71	59	
Age				0.525			0.817
≤60	195	28	167		107	88	
> 60	122	21	101		69	53	
Tumor size (cm)				0.004			0.000
< 5 cm	211	23	183		95	111	
≥ 5 cm	106	26	85		81	30	
Differentiation grade				0.193			0.451
Well	90	10	80		55	35	
Moderate	152	23	129		81	71	
Poor	75	16	59		40	35	
T stage				0.166			0.177
T1~T2	97	11	86		48	49	
T3~T4	220	38	182		128	92	
Lymph node status				0.460			0.119
Negative	167	25	142		87	80	
Positive	150	24	126		89	61	
Distant metastasis				0.010^b^			0.017
*M0*	291	40	251		156	135	
*M1*	26	9	17		20	6	
Perineural invasion				0.127^b^			0.232
Negative	299	44	255		168	131	
Positive	18	5	13		8	10	

^a ^Pearson χ^2 ^test

^b ^Adjusted χ^2 ^test

### Survival analysis

All patients underwent follow-up until cancer-related death or > 5 years after tumor resection. The 5-year survival of HER-2/neu-positive patients was 63.3%, and did not differ significantly from that of HER-2/neu-negative individuals (66.0%; *χ2 = 0.062, P = 0.804*). The 5-year survival for patients with negative and positive VEGF expression was 70.2% and 61.9% respectively, with a tendency of shorter survival in the latter versus former group, although the difference was not statistically significant (*χ^2 ^= 2.114, P = 0.146*).

## Discussion

HER-2/neu is a proto-oncogene that encodes a transmembrane protein with tyrosine kinase activity and is closely related to but biologically distinct from EGFR. Amplification and overexpression of the HER-2/neu gene have been demonstrated in several tumors such as breast, stomach, lung, and urinary bladder. The frequency of positivity appears to increase with clinical stage of disease and is associated with a worse prognosis [22-24]. Although Her-2/neu has been postulated as a prognostic biological marker for colorectal caner, conflicting data exist about the prevalence of HER-2/neu overexpression in CRC as well as its relationship with prognosis. We examined 317 colon cancer samples for the presence of Her-2/neu oncoprotein by immunohistochemistry. In all, 49 samples (15.5%) showed positive expression of Her-2/neu; only 7 samples were strongly membrane positive. Similar results were described in the study of Kavanagh et al.[25] They examined HER-2/neu protein expression in 132 CRC specimens, and found HER-2/neu overexpression in 11%, with 2 displaying were strong membranous immunostaining[25]. Our research showed that HER-2/neu expression was related with tumor size and distant metastases, suggesting that this protein may participate in tumor growth and distant metastasis although it was not a significant index of survival.

Previous reports that have demonstrated associations between HER-2/neu protein expression and more aggressive colorectal tumors. Demirbas et al [26] reported an association between HER-2/neu overexpression and tumor size (> 5 cm), differentiation grade, and vascular and lymphatic invasion. Besides, their patients with HER-2/neu protein-positive expression had shorter disease-free survival and overall survival compared with those who were negative. They suggested that overexpression of HER-2/neu protein played an important role in the progression of CRC and was considered an independent prognostic indicator. On the other hand, Jesus et al [27] reported that the HER-2/neu expression was not correlated with sex, age, tumor differentiation, localization of the primary tumor and overall survival. They indicated that HER-2/neu expression was unlikely to play a major role in the therapeutic management of colorectal cancer.

There are several possible reasons for discrepancies between studies to date. The most likely reason for the divergent findings is the different scoring systems-for example, Uner et al[28] judged only membrane staining in > 20% of tumor cells to be positive. The inclusion of cytoplasmic positivity in some papers may also be responsible for the conflicting results, because there is relatively high percent of positive cytoplasmic staining in CRC. Sensitivity of the antibodies used also makes the comparison between studies very challenging; the HercepTest™ kit (Dako) is the only antibody recommended by the FDA for HER-2/neu examination. Different antibodies may lead to different results, Park et al [23] reported a 47% protein expression rate using a polyclonal antibody (Zymed, South San Francisco, USA) and correlated overexpression with a higher incidence of postoperative recurrence. Conversely, Schuell et al [29] demonstrated an overexpression rate of 4% that was not correlated with survival using the validated HercepTest™ kit. Moreover, immunohistochemistry is a semiquantitative method and may easily be influenced by researchers' subjective perception. In addition, ethnic diversity of patients should be taken into account. Ghaffarzadegan et al[30] examined 69 Iranian colorectal samples and showed HER-2/neu staining in a high percent of cases with 65.9% showing cytoplasmic staining and 34.1% membranous-cytoplasmic staining and more prominent membranous staining in higher stages and grades. Kavanagh et al[25] detected that HER-2/neu protein was overexpressed in only 11% of Irish CRC patients, and found no correlation with tumor grade, Dukes' stage, time to recurrence and 5-year survival.

Angiogenesis represents an important event in the process of tumor invasion and metastases, and it is well established that VEGF is one of the most important molecules promoting endothelial cell migration, proliferation, and differentiation [31,32]. In our study, VEGF expression was noted in more than half of colon cancers (55.5%). Both the incidence and proportion of VEGF expression increased with the progression of colorectal carcinogenesis classified by depth of tumor invasion, presence of lymph node metastases and distant metastases, consistent with prior studies reported by Takahashi et al[33] and Kang et al[34]. Patients with positive VEGF expression had a tendency of shorter overall survival, although the difference versus negative patients was nonsignificant, suggesting that VEGF-positive phenotype in colon cancer may not provide additional prognostic value. This result is consistent with that of Lee et al [35]. In contrast, Kang et al [34] found that immunohistochemical expression of VEGF was an independent prognostic factor for CRC patients.

VEGF is one of the most potent inducers of angiogenesis, whereas HER-2/neu has been implicated in the regulation of VEGF. In human breast cancer, overexpression of HER-2/neu is correlated with increased VEGF expression [17,19]. Klos et al [20] demonstrated that HER-2/neu activation led to translational upregulation of VEGF and increased angiogenesis through ERK, PI3K/Akt, mTOR, and p70S6K. Petit et al [36] observed that neutralizing antibodies (trastuzumab) against HER-2/neu-positive breast cancer cell lines downregulated expression of VEGF by twofold. On the other hand, trastuzumab decreased tumor VEGF expression through the PI3K/Akt pathway in HER-2/neu-positive cancer cells[11,37-39], and could also increase anti-angiogenic factor and inhibit additional pro-angiogenic factors such as transforming growth factor-α, angiopoietin-1, plasminogen-activator inhibitor-1, and interleukin(IL)-8 [11,37]. Therefore, trastuzumab could exert synergistic interaction with anti-VEGF monoclonal antibody to suppress tumor angiogenesis by modulation of multiple angiogenic factors. Human breast cancers overexpressing HER-2/neu may be ideal targets for dual therapy with agents that inhibit VEGF and HER-2/neu. Conversely, no correlation was found between VEGF and c-erbB-2 in squamous cell carcinoma of the head and neck [40], and there is little research on correlation of VEGF and HER-2/neu in colon cancer.

The present study showed that VEGF was higher in HER-2/neu-positive tumor specimens than in those that were negative. Meanwhile, HER-2/neu expression was greater in VEGF-positive tumor than in VEGF-negative tumor, although the difference was not statistically significant. These findings suggest that there is no apparent correlation between HER-2/neu and VEGF expression in colon cancer. Our results are similar to those of Ochs et al [41] who reported that association was not supported between HER-2/neu and VEGF expression in stage II colon cancer. In breast cancer, angiogenesis could be regulated by HER-2 pathway even in the absence of HER-2/neu overexpression, targeting downstream targets of HER-2, particulary those leading to VEGF transcription and angiogenesis, could likely produce additional antitumor effects [42]. However, whether this approach could be useful against colon cancer neovascularization deserves further research.

## Conclusions

Our study aimed at evaluating the expression of c-erbB-2 and VEGF in colon cancer by immunohistochemical methods. We found HER-2/neu and VEGF is not important prognostic markers of colon cancer, and our results do not support an association between HER2/neu and VEGF expression.

## Competing interests

The authors declare that they have no competing interests.

## Authors' contributions

PC designed the study, performed the statistical analyses and wrote the manuscript.QGL collected clinicopathological data and performed laboratory analysis. JL, DRW, QGL scored the immunostained slides, prepared the images, and reviewed the manuscript. All authors read and approved the final manuscript.

## Pre-publication history

The pre-publication history for this paper can be accessed here:

http://www.biomedcentral.com/1471-2407/11/277/prepub
